# A multivariable miRNA signature delineates the systemic hemodynamic impact of arteriovenous shunt placement in a pilot study

**DOI:** 10.1038/s41598-020-78905-y

**Published:** 2020-12-11

**Authors:** Dominic Henn, Masood Abu-Halima, Mustafa Kahraman, Florian Falkner, Katharina S. Fischer, Janos A. Barrera, Kellen Chen, Geoffrey C. Gurtner, Andreas Keller, Ulrich Kneser, Eckart Meese, Volker J. Schmidt

**Affiliations:** 1 Hagey Laboratory for Pediatric Regenerative Medicine, Division of Plastic and Reconstructive Surgery, Stanford University School of Medicine, 257 Campus Dr. West, Stanford, CA 94305 USA; 2 BG Trauma Center Ludwigshafen, Heidelberg University, Ludwigshafen, Germany; 3 Institute for Human Genetics, Saarland University, Homburg, Germany; 4 Institute for Clinical Bioinformatics, Saarland University, Saarbrücken, Germany; 5 Department for Plastic and Breast Surgery, Zealand University Hospital Roskilde, Roskilde, Denmark

**Keywords:** Cardiovascular diseases, Skin diseases, Trauma

## Abstract

Arteriovenous (AV) fistulas for hemodialysis can lead to cardiac volume loading and increased serum brain natriuretic peptide (BNP) levels. Whether short-term AV loop placement in patients undergoing microsurgery has an impact on cardiac biomarkers and circulating microRNAs (miRNAs), potentially indicating an increased hemodynamic risk, remains elusive. Fifteen patients underwent AV loop placement with delayed free flap anastomosis for microsurgical reconstructions of lower extremity soft-tissue defects. N-terminal pro-BNP (NT-proBNP), copeptin (CT-proAVP), and miRNA expression profiles were determined in the peripheral blood before and after AV loop placement. MiRNA expression in the blood was correlated with miRNA expression from AV loop vascular tissue. Serum NT-proBNP and copeptin levels exceeded the upper reference limit after AV loop placement, with an especially strong NT-proBNP increase in patients with preexistent cardiac diseases. A miRNA signature of 4 up-regulated (miR-3198, miR-3127-5p, miR-1305, miR-1288-3p) and 2 down-regulated miRNAs (miR30a-5p, miR-145-5p) which are related to cardiovascular physiology, showed a significant systemic deregulation in blood and venous tissue after AV loop placement. AV loop placement causes serum elevations of NT-proBNP, copeptin as well as specific circulating miRNAs, indicating a potentially increased hemodynamic risk for patients with cardiovascular comorbidities, if free flap anastomosis is delayed.

## Introduction

**Figure 1:**
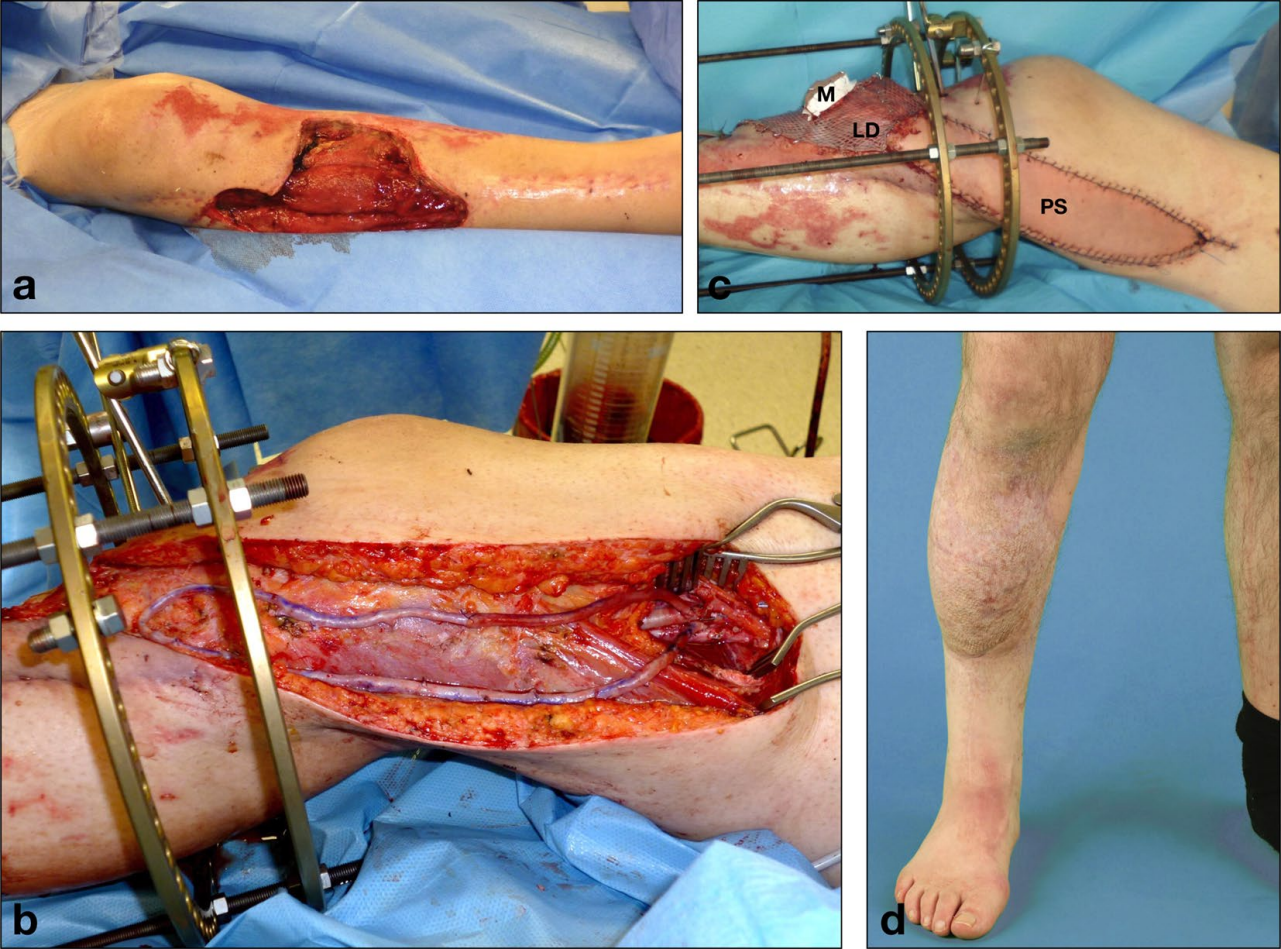
Clinical example of a 41 year-old patient who had sustained a traumatic lower leg soft-tissue defect and 3rd degree open tibial fracture (**a**). After debridement and placement of a cement spacer into the tibial defect and an Ilizarov external fixator by orthopedic surgery, an arteriovenous loop from the greater saphenous vein was placed and connected to the femoral artery (**b**), to which a combined latissimus dorsi (LD) and parascapular flap (PS) was then anastomosed, M = perforator-based monitor island (**c**). Stable defect coverage and good functional outcome at 1-year follow-up **(d)**.

The outcome of microsurgical reconstructions critically depends on the quality of local recipient vessels at the defect site. Atherosclerosis, trauma, infections, or oncologic resections can lead to compromised recipient vessels for free flap anastomoses, which necessitates the placement of interposition vein grafts or arteriovenous (AV) loops to connect flap pedicles to distant healthy vessels (Fig. 1)^1–3^. Free flap anastomoses to AV loops can be performed in one surgery or alternatively in a two-stage approach with comparable surgical outcomes^1^. Advantages of two-stage reconstructions include shorter operative times of the individual surgeries and the possibility of ultrasonographic diagnosis of early loop thrombosis before flap anastomosis, which may make a two-stage approach appear beneficial in patients with multiple comorbidities. However, the systemic impact of AV shunts in patients undergoing lower extremity reconstructions and especially their cardiovascular implications have not been investigated so far.

In hemodialysis patients, it has been shown that AV fistulas lead to an increased venous return and cardiac volume loading, which can lead to diastolic cardiac dysfunction and in some cases chronic congestive heart failure^2^. As a response to ventricular wall stretch due to cardiac volume loading, brain natriuretic peptide (BNP) and the N-terminal fragment of its precursor peptide (NT-proBNP) are released from cardiomyocytes into the circulation^3,4^. NT-proBNP and BNP are robust biomarkers with diagnostic and prognostic value for patients with heart failure, left ventricular dysfunction, and acute coronary syndromes^5,6^.

In recent years Copeptin, has been discussed as a novel biomarker for heart failure with a predictive value for all-cause mortality comparable to NT-proBNP. Copeptin is the C-terminal part of pro-Arginine Vasopressin (CT-proAVP) and is released together with AVP into the circulation with very stable plasma levels^7,8^. As with BNP and NT-proBNP, elevated levels of Copeptin have also been reported in patients with hemodialysis shunts^4^.

Recently, circulating microRNAs (miRNA) have been investigated as novel biomarkers in heart failure, with specific miRNA signatures showing an even better sensitivity for left ventricular dysfunction compared to traditional peptide markers such as NT-proBNP or BNP^9^.

The goal of our study was to determine the impact of two-stage AV loop reconstructions on the expression levels of circulating miRNAs and the serum levels of NT-proBNP and copeptin as established cardiac biomarkers, in order to provide guidance for risk stratification, selection of operative technique and timing in microsurgical reconstructions with AV loops. Moreover, we correlated the miRNA signatures from peripheral blood of AV loop patients with previously determined miRNA expression profiles from human venous AV loop tissue^10^ to identify systemic perturbations of miRNA expression after AV loop placement across different tissue types.

## Results

### Clinical outcomes

**Figure 2:**
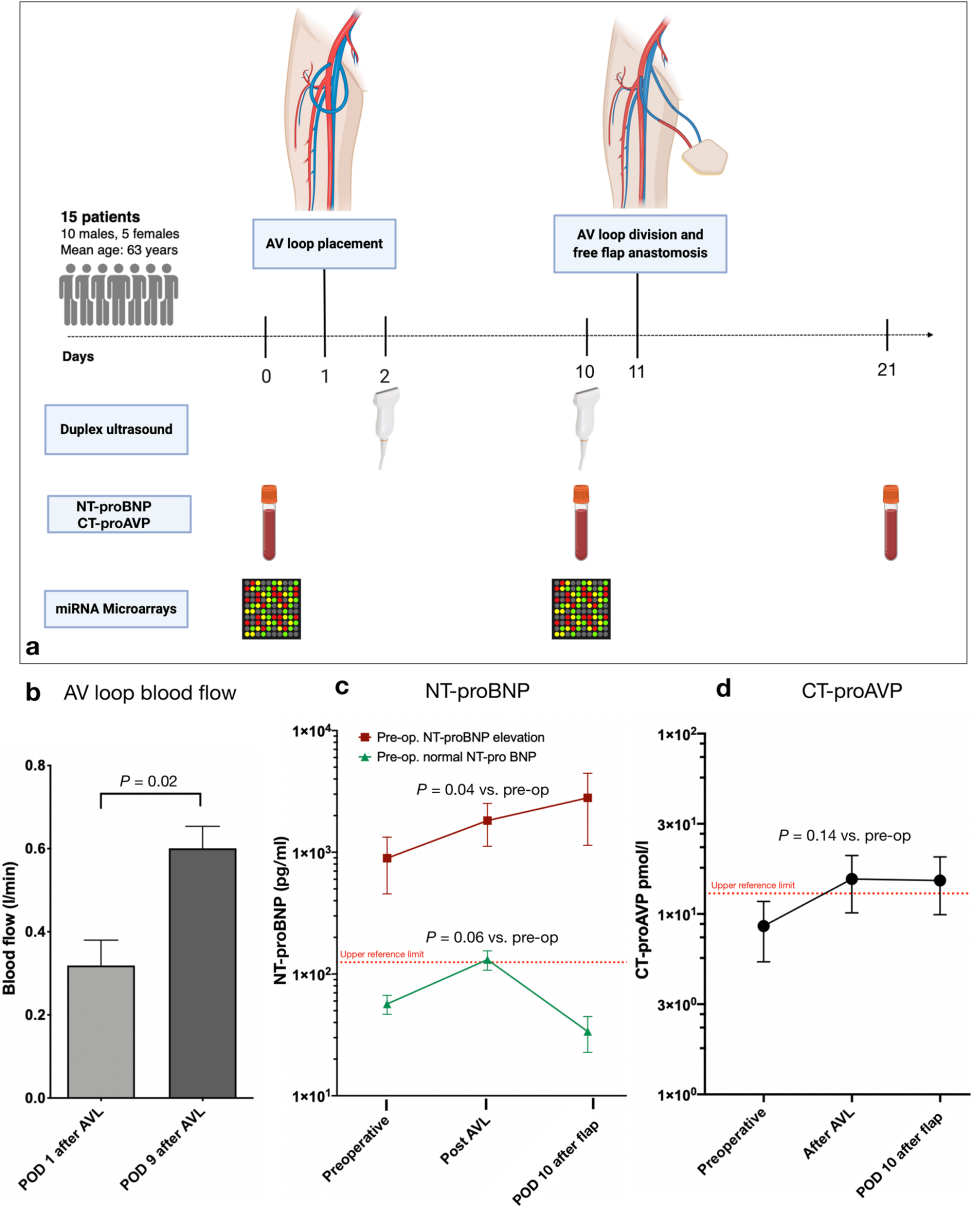
(**a**) Workflow of the study. AV loop = arteriovenous loop, NT-proBNP = N-terminal pro-brain natriuretic peptide. CT-proAVP (Copeptin) = (C-terminal part of pro-Arginine Vasopressin) (**b**) Comparison of mean blood flow in the AV loop as determined by duplex ultrasound on postoperative day (POD) 1 and 9 after AV loop placement. (**c**) Serum NT-proBNP concentration (y axis, logarithmic) in patients with pre-operatively (pre-op.) normal (green) or elevated (red) NT-proBNP levels. (**d**) Copeptin (C-terminal part of pro-Arginine Vasopressin) concentration; Preoperative = before AV loop placement; Post AVL = POD 9 after AV loop placement; POD 10 after flap = postoperative day 10 after flap anastomosis to the AV loop; * = p < 0.05. Figure schematic created with biorender.com.

The workflow of the study is shown in Fig. 2a. Fifteen patients (mean age: 63, range 36–84 years) underwent two-stage AV loop reconstructions between 2016 and 2018. In all cases recipient vessels in the proximity of the defect were inadequate and did not allow for primary free flap anastomosis. The patients’ demographic characteristics, defect etiologies, and flap types are summarized in Table 1. Overall, major complications requiring surgical intervention occurred in 7 patients (46%) (Table 2). Two patients developed a thrombosis of the AV loop prior to flap anastomosis on postoperative day (POD) 1 and 2 which was diagnosed by Doppler ultrasound and required emergent AV loop revision and thrombectomy. Both patients underwent free flap anastomosis to the AV loop uneventfully and recovered without further postoperative complications. One patient with a traumatic knee defect, which had been reconstructed with a latissimus dorsi flap, developed a complete thrombosis of the venous limb of the AV loop and flap pedicle after flap anastomosis. Emergent flap revision revealed diffuse microvascular damage. The flap could not be salvaged and the patient underwent above-knee amputation and prosthetic management. All patients were closely monitored by an internist for clinical signs of worsening heart failure or cardiac decompensation, which did not occur in any patient during their hospitalization. The medical management of all patients was also closely supervised by the patients’ cardiologists.

**Table 1:** Demographic characteristics, comorbidities and flap types of the patients.

Demographics	n (%)
**Gender**	
Female	5 (33)
Male	10 (67)
Mean age (range)	63 (36–84)
**Race**	
Caucasian	14 (93)
African American	1 (7)
**Comorbidities**	
Hypertension	6 (40)
CAD	3 (15)
MI	2 (13)
ICD	2 (13)
Valve replacement	2 (13)
PVD	4 (27)
DM	4 (27)
History of tobacco use	1 (6)
CKD	3 (15)
Coagulation disorder	2 (13)
**Defect etiology**	
Trauma	7 (47)
Infection	3 (20)
Tumor	2 (13)
Ulcer	1 (6)
Unstable scarring	1 (6)
**Defect location**	
Lower leg	8 (53)
Knee	1 (6)
Foot	2 (13)
Pelvis	4 (27)
**Flap type**	
LD	10 (67)
Parascapular	1 (6)
Combined LD and Parascapular	2 (13)
Rectus abdominis	2 (13)

*CAD* coronary artery disease, *ICD* implanted cardioverter defibrillator, *MI* myocardial infarction, *PVD* peripheral vascular disease, *DM* diabetes mellitus, *CKD* chronic kidney disease, *LD* latissimus dorsi muscle flap.

**Table 2:** Postoperative complications of the patients.

Postoperative Complications	n (%)
AV loop thrombosis before flap anastomosis	2 (13)
Thrombosis after flap anastomosis	1 (6)
Minor wound complication	1 (6)
Major wound complication	5 (33)
Total flap failure	1 (6)
Hematoma recipient site	3 (20)
Hematoma donor site	1 (6)
Wound complication donor site	1 (6)

*AV* arteriovenous.

### AV loop blood flow

Blood flow in the AV loop was measured on the first postoperative day after AV loop placement and on the day before flap anastomosis to the loop using duplex ultrasound. Immediately after AV loop creation a mean blood flow of 0.31 l/min was measured, which almost doubled by the time of flap anastomosis to an average flow of 0.60 l/min (1.94-fold increase; p < 0.05) (Fig. 2b).

### NT-proBNP

Peripheral venous blood sampling was performed 1 day before AV loop placement (baseline), on the day before flap anastomosis, and on POD 10 after flap anastomosis. The patient cohort of our study had a markedly high rate of preexistent cardiovascular disease with 53% of patients being affected by coronary artery disease, myocardial infarction, heart failure with implantable cardioverter defibrillator (ICD), cardiac valve replacement, or arterial hypertension (Table 1). Thus, the preoperative average NT-proBNP level of all patients was 636.0 pg/ml, which is far above the laboratory’s normal upper reference limit of 100 pg/ml. After AV loop placement, a significant and almost twofold increase in mean NT-proBNP to 1233.36 pg/ml was measured (1.93-fold increase, p < 0.05). Nine patients (60%) had elevated NT-proBNP levels preoperatively (mean: 893.44 pg/ml), which increased more than two-fold after AV loop placement (mean: 1819.78 pg/ml, p < 0.05). Patients with normal preoperative NT-proBNP levels (mean: 56.75 pg/ml) also showed an average increase to 131.25 pg/ml, which is above the normal upper reference limit. This, however, was not statistically significant and normalized by postoperative day 10 after AV loop placement. By contrast, NT-proBNP further increased by POD 10 (mean: 2799 pg/ml) in patients with a pre-operative NT-proBNP elevation (Table 3, Fig. 2c).

**Table 3:** N-terminal pro brain natriuretic peptide (NT-pro BNP) levels of the patients.

NT-pro BNP (pg/ml)	Preoperative	Post-AVL placement	POD 10 after flap anastomosis	*P*-value^1^	*P*-value^2^	*P*-value^3^
All patients (n = 15) (mean ± SEM)	636.0 (317.77)	1233.36 (419.03)	1885.0 (1098.29)	0.03	0.39	0.23
Pre-op. NT-proBNP elevation (n = 9; mean ± SEM)	893.44 (438.0)	1819.78 (698.88)	2799.0 (1656.77)	0.04	0.42	0.23
Pre-op. normal NT-proBNP (n = 6; mean ± SEM)	56.75 (9.99)	131.25 (23.76)	33.75 (11.04)	0.06	0.06	0.16

AVL = arteriovenous loop, POD = postoperative day.

^1^T-Test *Preoperative vs. Post-AVL placement.*

^2^T-Test *Post AVL placement vs. POD 10 after flap anastomosis.*

^3^T-Test *Preoperative vs. POD 10 after flap anastomosis.*

### Copeptin (CT-proAVP)

The mean pre-operative copeptin level in our patient cohort was 8.58 pmol/l and thus within the normal references rage. After AV loop placement, copeptin levels increased to 15.63 pmol/l and thus exceeded the laboratory’s upper reference limit (13 pmol/l). Due to the limited sample size of our study the comparison between copeptin levels before and after AV loop placement missed statistical significance, however there was a trend toward higher copeptin levels after AV loop placement (p = 0.12). In accordance with the observed increase in NT-proBNP levels, copeptin levels remained elevated at POD 10 after flap anastomosis to the AV loop (15.33 pmol/l) (Table 4, Fig. 2d).

**Table 4:** Copeptin (CT-proAVP) levels of the patients.

Copeptin (CT-proAVP) (pmol/l)	Preoperative	Post-AVL placement	POD 10 after flap anastomosis	*P-value*^1^	*P-value*^2^	*P-value*^3^
All patients (n = 15) (mean ± SEM)	8.58 (3.14)	15.63 (5.47)	15.33 (5.48)	0.15	0.89	0.20

AVL = arteriovenous loop, POD = postoperative day.

^1^T-Test *Preoperative vs. Post-AVL placement.*

^2^T-Test *Post AVL placement vs. POD 10 after flap anastomosis.*

^3^T-Test *Preoperative vs. POD 10 after flap anastomosis.*

### MicroRNA expression analysis

**Figure 3:**
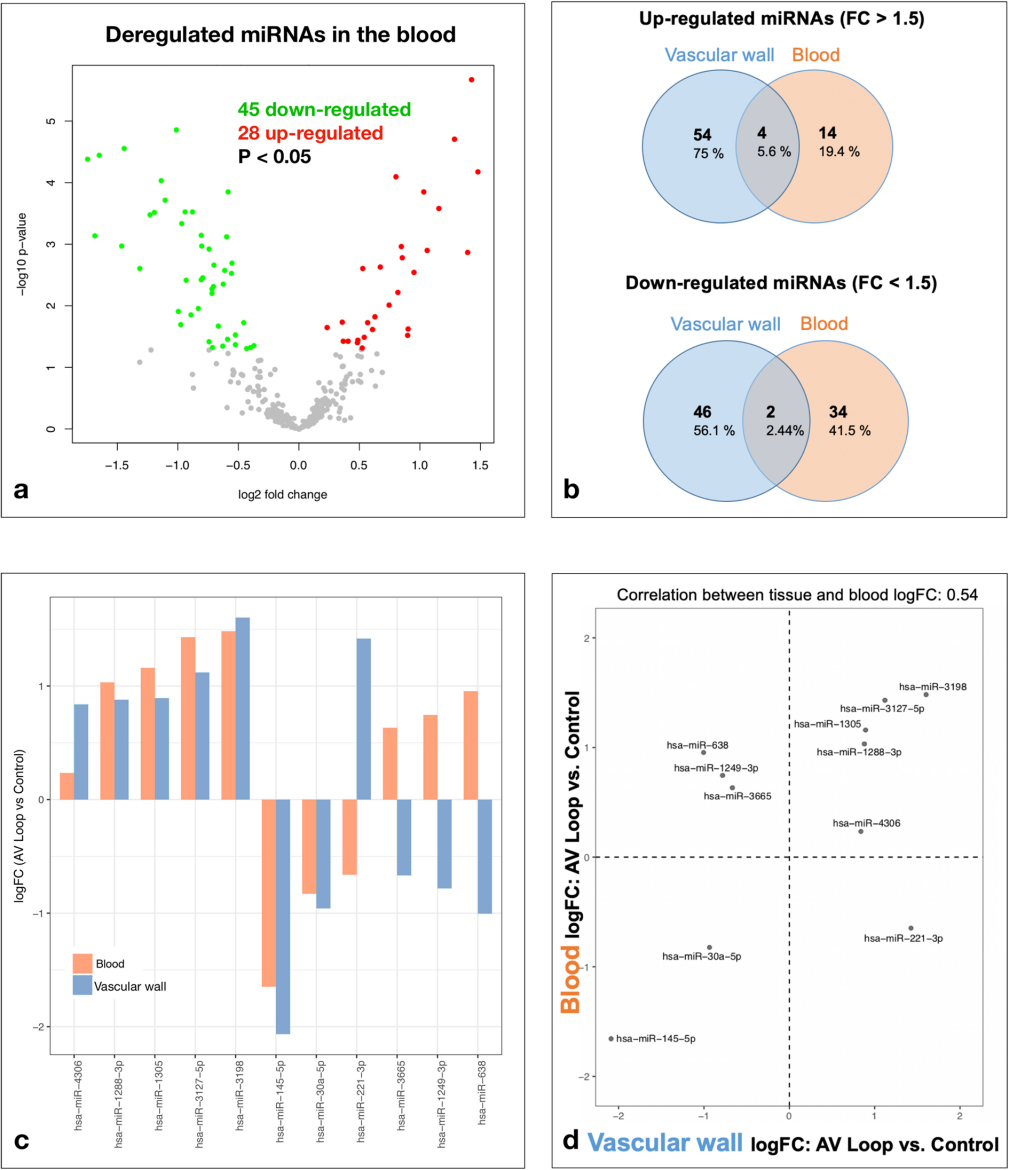
(**a**) Volcano plot showing the deregulated miRNAs in the blood on postoperative day (POD) 9 after arteriovenous (AV) loop placement. Colored dots represent significantly deregulated miRNAs (p < 0.05) with red dots representing up-regulated and green dots representing down-regulated miRNAs. (**b**) Venn diagram showing the absolute and relative count of miRNAs up- and down-regulated by fold-change (FC) > 1.5, resp. < 1.5 in the peripheral blood and the vascular wall of AV loops (POD 10 after loop placement). The complete miRNA expression profiles in vascular AV loop tissue were previously published^10^. (**c,d**) Correlation between miRNA expression profiles in blood and vascular tissue reveals 5 concordantly up-regulated miRNAs (**d**, upper right quadrant), 2 concordantly down-regulated miRNAs (**d,** lower left quadrant), and 4 discordantly deregulated miRNAs (**d**, upper left and lower right quadrants).

Peripheral venous blood samples collected before and after AV loop placement using PAXgene Blood RNA tubes (Qiagen, Hilden, Germany) were subjected to RNA isolation and miRNA microarrays. Microarray analysis revealed a significantly deregulated expression of 73 miRNAs with 45 being down-regulated and 28 miRNAs being up-regulated compared to their pre-operative expression levels (raw p value < 0.05) (Fig. 3a). Among these deregulated miRNAs, 7 miRNAs were significantly up-regulated with a log_2_ fold-change > 1 and 11 miRNAs were significantly down-regulated with a log_2_ fold-change < 1(adjusted p value < 0.05) (Table 5).

**Table 5:** The miRNA expression determined by miRNA microarrays.

miRNA	Raw *P* value	Adjusted *P* value	FC	Log_2_ FC	Expression change
hsa-miR-1288-3p	0.000140	0.004	2.045	1.032	Up
hsa-miR-1207-5p	0.001243	0.013	2.087	1.061	Up
hsa-miR-1305	0.000261	0.005	2.233	1.159	Up
hsa-miR-3125	0.000020	0.002	2.437	1.285	Up
hsa-miR-564	0.001347	0.014	2.631	1.396	Up
hsa-miR-3127-5p	0.000002	0.001	2.695	1.430	Up
hsa-miR-3198	0.000067	0.003	2.794	1.482	Up
hsa-miR-186-5p	0.000041	0.002	0.299	− 1.742	Down
hsa-miR-513a-5p	0.000718	0.010	0.311	− 1.685	Down
hsa-miR-145-5p	0.000036	0.002	0.319	− 1.648	Down
hsa-miR-1260a	0.001063	0.012	0.363	− 1.463	Down
hsa-miR-513b-5p	0.000028	0.002	0.368	− 1.442	Down
hsa-miR-942-5p	0.002462	0.021	0.403	− 1.311	Down
hsa-miR-15b-3p	0.000328	0.005	0.427	− 1.227	Down
hsa-miR-4318	0.000301	0.005	0.437	− 1.193	Down
hsa-miR-4317	0.000093	0.003	0.455	− 1.136	Down
hsa-miR-550a-5p	0.000193	0.004	0.466	− 1.102	Down
hsa-miR-106b-3p	0.000014	0.002	0.497	− 1.008	Down

*FC* fold-change.

### Confirmation of miRNA expression by RT-qPCR

Microarray data was confirmed by real-time quantitative PCR (RTqPCR), which showed a significant upregulation of the 5 selected miRNAs (miR-1231, miR135b-3p, miR-3127, miR-3198, miR-519e-5p) in the peripheral blood after AV loop placement (p < 0.05) (Table 6).

**Table 6:** The miRNA expression determined by real-time quantitative PCR (RT-qPCR).

Target	FC	P value	Regulation
miR-1231	1.87	0.005	Up
miR-3127	1.74	0.02	Up
miR-3198	2.0	0.005	Up
miR-135b-3p	2.0	0.004	Up
miR-519e-5p	2.0	0.007	Up

*FC* fold-change.

### Correlation of miRNA expression in peripheral blood and AV loop vascular tissue

In order to identify common miRNA signatures in tissue and blood after AV loop placement, we then correlated the microarray expression profiles obtained from the peripheral blood samples with previously determined miRNA expression profiles from tissue samples of the venous wall from AV loops in patients undergoing lower extremity reconstructions^10^, and found 207 miRNAs expressed in both blood and venous tissue. When taking only the significantly deregulated miRNAs into account, a positive correlation was found between miRNA expression in blood and vascular tissue (r = 0.54). We identified a miRNA signature of 4 up-regulated miRNAs, namely miR-3198, miR-3127-5p, miR-1305, miR-1288-3p and 2 down-regulated miRNAs, namely miR30a-5p and miR-145-5p, showing a significant deregulation (p < 0.05, FC < or > 1.5) in blood and venous tissue (Fig. 3b–d).

### miRNA enrichment and annotation analysis

**Figure 4:**
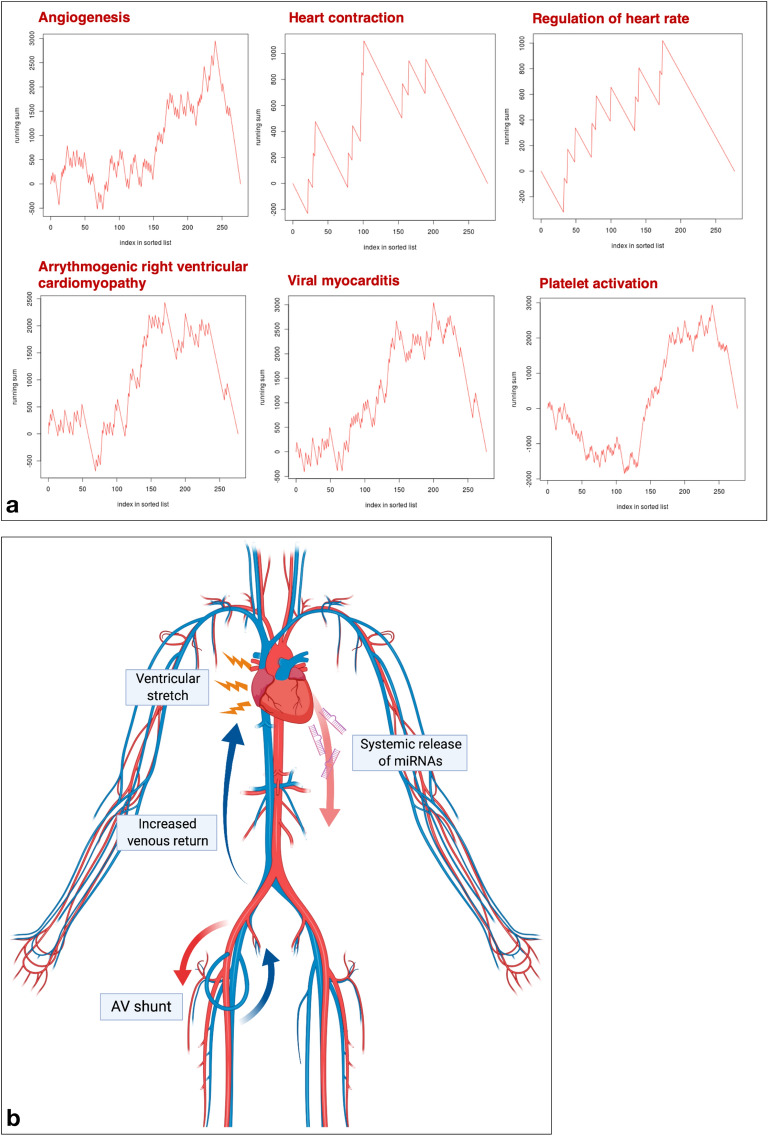
**(a)** Selection of gene sets with predicted enrichment in response to the deregulated miRNAs in the peripheral blood after arteriovenous (AV) loop placement by miRNA Enrichment and Annotation Analysis. (**b**) Pathophysiologic mechanism behind systemic miRNA release into the circulation. AV loops cause an increase in venous return to the heart, leading to cardiac volume loading and ventricular stretch, which is a stimulus for the release of natriuretic peptides and likely also miRNAs into the circulation. Figure schematic created with biorender.com.

To identify gene expression pathways which are affected by the deregulated miRNAs in the peripheral blood, miRNA Enrichment and Annotation Analysis (miEAA) was performed^11^. We found 652 gene sets which were predicted to be significantly enriched and 341 gene sets predicted to be significantly depleted in response to the observed miRNA deregulations in the blood after AV loop placement. Among these gene sets, 14 enriched and 3 depleted gene sets were associated with cardiac function, cardiovascular disease, and angiogenesis (Table 7, Fig. 4a).

**Table 7:** Enriched and depleted gene sets associated with cardiac function, cardiovascular disease, and angiogenesis.

Category	Subcategory	*P* value	Number of miRNAs
**Enriched gene sets**			
Gene Ontology	GO0001525 angiogenesis	0.026	105
Gene Ontology	GO0045765 regulation of angiogenesis	0.037	31
Gene Ontology	GO0019722 calcium mediated signaling	0.040	30
Gene Ontology	GO0030168 platelet activation	0.031	120
Gene Ontology	GO0090330 regulation of platelet aggregation	0.049	5
Gene Ontology	GO0055008 cardiac muscle tissue morphogenesis	0.038	10
Gene Ontology	GO0055015 ventricular cardiac muscle cell development	0.004	13
Gene Ontology	GO0055008 cardiac muscle tissue morphogenesis	0.038	10
Gene Ontology	GO0010460 positive regulation of heart rate	0.044	10
Gene Ontology	GO0060047 heart contraction	0.038	11
WikiPathways	WP1559 TFs Regulate miRNAs related to cardiac hypertrophy	0.048	24
KEGG	hsa05412 Arrhythmogenic right ventricular cardiomyopathy ARVC	0.034	62
KEGG	hsa05416 Viral myocarditis	0.012	80
**Depleted gene sets**			
Gene Ontology	GO2000727 positive regulation of cardiac muscle cell differentiation	0.024	17
Gene Ontology	GO0090050 positive regulation of cell migration involved in sprouting angiogenesis	0.041	33
Gene Ontology	GO0001946 lymphangiogenesis	0.033	26

## Discussion

Over the last few decades, technical advances in surgical instruments and monitoring devices for microsurgery as well as the growing body of literature on reliable donor sites for free flaps have enabled surgeons to perform personalized, safe, and successful microsurgical reconstructions, almost independent of a patient’s age^12,13^. However, with a rapidly aging population, and the fact that patients with higher American Society of Anaesthesiology (ASA) class have increased medical complications after free tissue transfer^14^, preoperative risk stratification and meticulous surgical planning adapted to a patient’s individual comorbidities becomes imperative. In patients with systemic atherosclerosis and peripheral vascular disease, recipient vessels at the defect site are often unsuitable for microvascular anastomosis. In these cases, AV loops are an established and highly versatile technique to connect flap pedicles to distant healthy vessels^1,15,16^.

We have previously shown that elevated vascular shear stress in AV loops leads to distinct miRNA signatures within the vascular wall^10^. MicroRNAs (miRNAs) are small non-coding RNAs which modulate the expression of about 60% of all protein-coding genes on the post-transcriptional level^17^. In recent years, significant efforts have been made to, identify circulating or tissue-specific changes in miRNA expression as novel biomarkers for a wide range of clinical conditions, including multiple cardiovascular diseases. It has been shown that, as opposed to single miRNAs, which are often not disease-specific, changes in the expression patterns of several miRNAs (miRNA signatures) can improve diagnostic sensitivity and specificity^18^. The authors (E.M. and A.K.) have previously shown that systolic heart failure causes deregulations of specific miRNA expression patterns, thus enabling the identification of a miRNA signature with high diagnostic potential for systolic heart failure^9^.

Recent studies have shown that 43% of patients with hemodialysis shunts develop chronic complications of heart failure due to persistently increased cardiac volume loading related to AV fistulae^19^. Here, we sought to determine the impact of AV loop placement in the lower extremity on circulating miRNA expression profiles and NT-proBNP as a standard biomarker for heart failure, to determine whether patients undergoing microsurgery with delayed flap anastomosis to AV loops might be at an increased risk for hemodynamic complications.

An average blood flow of 0.31 ml/min was measured on POD 1 after AV loop placement, which doubled to 0.6 ml/min until the day before flap anastomosis, which is in the range of flow rates reported for mature hemodialysis AV fistulas in the upper extremities^20^. The increase in blood flow over time is due to a reactive vascular dilation in response to elevated shear stress and mediated by nitric oxide (NO) released from endothelial cells (ECs)^21,22^. Apart from NO, the exposure of ECs to elevated shear forces leads to an upregulation of pro-angiogenic signaling pathways, as we have previously shown on AV loop venous tissue, which are also involved in the vascular remodeling process^10^.

An increase in serum NT-proBNP and copeptin compared to the pre-operative baseline levels was measured at the time of flap anastomosis to the AV loop. In patients with a pre-operatively elevated NT-proBNP due to cardiovascular comorbidities, a significant increase in serum NT-proBNP was measured, which remained elevated at POD 10 after flap anastomosis. Interestingly, in patients with a normal pre-operative NT-proBNP, the average NT-proBNP 9 days after AV loop placement also exceeded the upper reference limit, which, however, normalized by POD 10 after flap anastomosis. An inability to compensate for volume loading during free flap surgeries might have contributed to the persistent elevation of NT-proBNP and copeptin levels 10 days after loop division and flap anastomosis in patients with cardiovascular comorbidities.

Our findings indicate that not only long-standing hemodialysis AV shunts, but also short-term shunts in the lower extremity in patients undergoing reconstructive microsurgery can have a significant hemodynamic impact, leading to an increase in NT-proBNP and copeptin, especially in patients with cardiovascular comorbidities. The elevation of these biomarkers is a consequence of ventricular stretch due to an increased venous return to the heart^3^. Studies in patients with dialysis shunts have shown that AV fistula creation results in an immediate decrease in systemic vascular resistance^23^. The resulting acute fall in central and peripheral blood pressure leads to a reactive sympathetically mediated increase in heart rate, stroke volume and cardiac output^24^. Increased venous return can lead to right ventricular dilation and a rise in right atrial, pulmonary artery, and left ventricular end diastolic pressures, which can ultimately result in right ventricular dysfunction, pulmonary hypertension, and left ventricular hypertrophy^19,25^. Since these chronic cardiovascular complications are the result of long-standing AV shunts in hemodialysis patients, short-term AV loops in the lower extremity in patients requiring microsurgical reconstructions likely do not pose a risk for the development of chronic heart failure. However, elevated serum NT-proBNP and copeptin levels clearly indicate that AV loops have an acute hemodynamic impact on ventricular physiology, especially in patients with preoperative cardiovascular comorbidities. In light of the relatively high prevalence of cardiovascular morbidity due to systemic atherosclerosis in patients requiring AV loop placements in order to achieve successful microsurgical defect reconstruction, preoperative consideration should be given to systemic hemodynamic effects of temporary AV loop placement.

We have previously shown that one-stage AV loop reconstructions can be performed at comparable postoperative surgical complication rates as two-stage reconstructions, but require significantly longer operative times^1^. In light of the results of our current study, risks and benefits of both approaches have to be carefully assessed when developing and individualized reconstructive strategy for each patient, taking a patient’s comorbidities and the systemic hemodynamic impact of AV loop placement on pre-existing cardiac disease into account.

Using miRNA transcriptomic profiling with microarrays, we have identified a miRNA signature that is systemically deregulated after AV shunt placement and can be detected in the peripheral blood as well as venous AV loop tissue. This signature consists of 4 up-regulated miRNAs (miR-3198, miR-3127-5p, miR-1305, miR-1288-3p) and 2 down-regulated miRNAs (miR30a-5p, miR-145-5p) which were significantly and strongly deregulated. As it has previously been shown for patients with heart failure^9^ and a variety of other clinical conditions^26–28^, circulating miRNA signatures have a strong diagnostic potential. Thus, the miRNA signature identified here might serve as valuable diagnostic tool for quantification of the systemic hemodynamic impact of vascular shunts in dialysis patients or patients undergoing microsurgical reconstructions in the future. This, however, will require a correlation with clinical hemodynamic parameters and confirmation of our findings in a larger patient population in future studies.

The clinical significance of our findings is underscored by the fact that the systemically up-regulated miRNAs in the peripheral blood of patients with AV loops have been related to a variety of cardiovascular diseases in previous studies. Studies on mouse and human heart failure have found an up-regulation of miR-638 which was also significantly up-regulated in the blood after AV loop placement^29^. Abundant levels of miR-1249, which were found in the blood of patients with AV loops, have also been detected in patients with cardiac myxoma^30^ and have been related to myocardial apoptosis^31^. MiRNAs have also been linked to congenital cardiac conditions such as Marfan syndrome^32^ and hypoplastic left heart syndrome, which is associated with a deregulated expression of miR-1305^33^, for which an up-regulation was found in the blood and vascular tissue of AV loop patients. MiR-4306 has been suggested as a biomarker for patients with acute aortic dissection^34^, and was also found to be up-regulated in blood and vascular tissue of patients in our study. An up-regulation of miR-221 was only found in AV loop vascular tissue and has been previously identified to induce angiogenesis and regulate endothelial cell biology^35^, which is in line with our previous findings of up-regulated pro-angiogenic signaling pathways in vascular tissue of patients with AV loops and in experimental AV loop rat models (Fig. 4b)^10,36^.

Bioinformatic prediction of affected gene sets in response to the deregulated miRNAs identified in the blood of AV loop patients further confirmed our findings and showed an enrichment of gene sets related to hemodynamic changes in response to AV shunt flow (GO0060047: heart contraction, GO0010460 positive regulation of heart rate) as well as cardiac pathologies such as viral myocarditis (Kyoto Encyclopedia for Genes and Genomes (KEGG): hsa05416)^37^ or cardiac hypertrophy (WikiPathways (WP): 1559). Moreover, the gene set for arrhythmogenic right ventricular cardiomyopathy (ARVC, KEGG: hsa05412) was significantly enriched, which indicates right ventricular involvement, likely due to increased venous return after AV loop placement.

Limitations of our study are mainly related to its small sample size. Larger patient cohorts would be required to achieve an adequate power to correlate biomarker levels to postoperative outcomes. However, given the findings of our pilot study, and previous reports of decompensated heart failure after hemodialysis shunt placement^2^, it seems very likely that a delay in flap anastomosis to an AV loop in patients with cardiovascular comorbidities would expose patients to an increased risk for cardiovascular complications, which might be avoided by one-stage AV loop placement and immediate flap anastomosis.

Further studies may correlate our findings with specific echocardiographic parameters; however, existing data from patients with dialysis shunts are in line with our findings and have provided the pathophysiologic background and echocardiographic correlates to the observed NT-proBNP and copeptin elevation in response to AV shunt placement^3^.

A further limitation of our study is the potential impact of demographic factors and comorbidities on miRNA expression. For the signature of 6 miRNAs, for which we have identified a systemic deregulation in response to AV shunt placement, previous studies have reported associations to a variety of tumor conditions^38–42^, and diabetes mellitus (miR-145-5p)^43^. None of our patients had a tumor diagnosis and diabetes mellitus was only present in 27% of all cases. Thus, given its low prevalence in our patient cohort, a systemic impact of diabetes on miR-145-5p expression seems unlikely.

Our data has relevant implications for clinical practice and pre-operative evaluation of patients with compromised vessels for reconstructive microsurgery. We recommend that, in addition to taking a detailed history with regard to cardiovascular comorbidities, serum NT-proBNP concentrations are determined in all patients evaluated for AV loop reconstructions. In cases of known cardiovascular comorbidities or preoperatively elevated NT-proBNP levels, one-stage reconstructions with immediate loop division and flap anastomosis might be preferable. However, longer operating times and increased perioperative stress in one-stage reconstructions have to be weighed against potential benefits.

If two-stage reconstructions are decided for, the time between loop placement and flap anastomosis should be shorter than 10 days in order to limit the hemodynamic impact of shunt flow as well as the risk for AV loop thrombosis, which, as we have previously shown, increases with longer time intervals^1^.

## Conclusion

AV loop placement in the lower extremity leads to elevated serum levels of NT-proBNP and a systemic deregulation of miRNAs, which are involved in cardiac function and diseases, in the peripheral blood and vascular tissue. These findings indicate that patients with cardiovascular comorbidities might be at risk for hemodynamic complications if flap anastomosis to an AV loop is delayed.

## Methods

### Patients

The study was conducted in accordance with the Declaration of Helsinki and was approved by the locally appointed ethics committee (832.259.15, Ethik-Kommission bei der Landesärztekammer Rheinland-Pfalz). All patients who were included in the study had provided written informed consent. All surgeries were performed at BG Trauma Center Ludwigshafen at Heidelberg University under general inhalation anesthesia. Our postoperative standard monitoring protocol for patients with AV loop reconstructions has been described previously and was followed in all patients^1^. A two-stage approach for flap anastomosis to the AV loop was decided for due to the multiple comorbidities and frailty of the patient population as well as the high complexity of the defects. The mean time between AV loop placement and flap anastomosis was 11.36 days (range 6–18 days). Wound infections and wound dehiscence without the need for surgical revision were classified as minor wound complications. Wound complications requiring surgical intervention, as well as partial flap necroses (partial necrosis of a skin island of a flap of > 5%) were defined as major wound complications.

### Duplex sonography and blood collection

Duplex ultrasound was performed using an Esaote MyLab alpha with an SL1543 probe (Esaote S.p.A., Genoa, Italy). NT-pro BNP and copeptin concentrations were determined with standard assays in the routine clinical chemistry laboratory of Ludwigshafen Hospital. To determine circulating levels of miRNAs, separate blood samples were collected using Paxgene Blood RNA tubes (Qiagen, Hilden, Germany).

### Sample preparation and RNA isolation

Total RNA including miRNAs was isolated from blood samples using the PAXgene Blood miRNA Kit (Qiagen) following the manufacturer’s recommendations, including DNase I treatment (Qiagen). The concentration of the isolated total RNA was measured using a NanoDrop ND-2000 spectrophotometer (Thermo Fisher Scientific, Waltham, MA). RNA purity was determined by OD (optical density) 260/280, which was between 1.9 and 2.1 in all samples. RNA integrity was assessed with the Agilent 6000 Nano Kit and an Agilent 2100 Bioanalyzer (Agilent, Santa Clara, CA).

### miRNA microarray analysis

For miRNA microarray analysis, SurePrint 8X60K Human v16 miRNA microarrays (Agilent) were used according to the manufacturer’s recommendations. Per sample, a total of 100 ng RNA was dephosphorylated using calf intestinal phosphatase (CIP) at 37 °C for 30 min and denatured with 100% dimethyl sulfoxide (DMSO) at 100 °C for 7 min. SureHyb chambers (Agilent) were used for assay hybridizations. The microarrays were washed, dried and scanned at a resolution of 3 μm double-pass using an Agilent G2565C Microarray Scanner. Data acquisition was performed using the AGW Feature Extraction software version 10.10.11 (Agilent).

### miRNA analysis by RT‑qPCR

The StepOnePlus Real-Time PCR System (Applied Biosystems, Foster City, CA) and the *mi*Script PCR System (Qiagen) were used to perform RT-qPCR validation of miRNA expression. All steps were performed according to the manufacturer’s instructions. The same samples used for microarray analysis were also used for RT-qPCR validation. Five miRNAs (miR-1231, miR-3127, miR-3198, miR-135b3p, miR-519e-5p) were chosen for RT-qPCR based on the significance of their abundance (highest fold-change) and biological significance in cardiovascular diseases^9,44^. Of the total RNA, 250 ng were converted into complementary DNA (cDNA). The resulting cDNA was diluted to 0.5 ng/μL, which was used for miRNA expression analysis. All PCR experiments were carried with the Liquid Handling Robot QIAgility (Qiagen). Primer assays were obtained from Qiagen. A miRNA reverse transcription control (miRTC) (Qiagen) was performed to assess the performance of the reverse transcription reaction. The melting curve analysis was used to determine the specificity of RT-qPCR products.

### Bioinformatics analysis

Statistical analysis was performed with R (versions 3.3.2 and 3.3.3; www.r-project.org). Raw data generated by the Agilent Feature Extraction software were quantile normalized, and signal intensities of miRNAs were log_2_-transformed. Only miRNAs detected in at least 75% of all samples were used for analysis. The *p* values were corrected for multiple testing using the Benjamini–Hochberg procedure. The DataAssist Software v3.0 (Applied Biosystems) was used to calculate the fold-changes in miRNA expression by the equation 2^−∆∆CT^ for RT-qPCR data. Correlation of blood and tissue microarray datasets was performed with the Pearson correlation. *P* values < 0.05 were considered statistically significant**.** MiRNA Enrichment and Annotation Analysis (MiEAA), a web based application (http://www.ccb.uni-saarland.de/mieaa_tool/) developed by the authors (E.M., A.K.)^11^, was used to perform miRNA Set Enrichment Analysis and over-representation analysis (ORA). Figures 2a and 4b were created with biorender.com.
